# Numerical Simulations of MREIT Conductivity Imaging for Brain Tumor Detection

**DOI:** 10.1155/2013/704829

**Published:** 2013-04-29

**Authors:** Zi Jun Meng, Saurav Z. K. Sajib, Munish Chauhan, Rosalind J. Sadleir, Hyung Joong Kim, Oh In Kwon, Eung Je Woo

**Affiliations:** ^1^Department of Biomedical Engineering, Impedance Imaging Research Center (IIRC), Kyung Hee University, Yongin, Republic of Korea; ^2^Department of Biomedical Engineering, University of Florida, Gainesville, FL, USA; ^3^Department of Mathematics, Konkuk University, Seoul, Republic of Korea

## Abstract

Magnetic resonance electrical impedance tomography (MREIT) is a new modality capable of imaging the electrical properties of human body using MRI phase information in conjunction with external current injection. Recent *in vivo* animal and human MREIT studies have revealed unique conductivity contrasts related to different physiological and pathological conditions of tissues or organs. When performing *in vivo* brain imaging, small imaging currents must be injected so as not to stimulate peripheral nerves in the skin, while delivery of imaging currents to the brain is relatively small due to the skull's low conductivity. As a result, injected imaging currents may induce small phase signals and the overall low phase SNR in brain tissues. In this study, we present numerical simulation results of the use of head MREIT for brain tumor detection. We used a realistic three-dimensional head model to compute signal levels produced as a consequence of a predicted doubling of conductivity occurring within simulated tumorous brain tissues. We determined the feasibility of measuring these changes in a time acceptable to human subjects by adding realistic noise levels measured from a candidate 3 T system. We also reconstructed conductivity contrast images, showing that such conductivity differences can be both detected and imaged.

## 1. Introduction

Brain tumors are serious and life-threatening because of their invasive and infiltrative characteristics [1]. Medical imaging plays a central role in the diagnosis of brain tumors [2, 3]. MRI is the preferred imaging modality for brain tumor diagnosis, providing detailed information of lesion type, size and location [4]. Although gadolinium-enhanced *T*1-weighted images and *T*2-weighted images are the MRI modalities of choice for the initial assessment, their usefulness in identifying tumor types, distinguishing tumors from nontumoral lesions, and assessing treatment effects is limited [4, 5]. For this reason, these scans may be used in combination with other advanced MRI techniques [5]. However, there is still a demand for new MR-based methods that can both detect and characterize brain lesions.

Magnetic resonance electrical impedance tomography (MREIT) is a technique that uses MRI to measure the internal magnetic flux density induced by externally injected currents [6–8]. Since the magnetic flux density perturbs the main field of an MRI scanner, one can obtain the *z*-component of the induced magnetic flux density (*B*_*z*_) by rescaling MR phase images [9–11]. Applying a conductivity image reconstruction algorithm [12–14], we can reconstruct high-resolution high-contrast conductivity image of the object. MREIT has been steadily developed from simulations, reconstruction algorithms, and imaging experiments using both phantoms and animals [12–17]. It has now reached a stage of *in vivo* human imaging experiments, and Kim et al. [18] recently reported the first such trial. Use of MREIT has also been suggested for neural activity detection in small-scale isolated neural structures [19] or as a means of understanding the effects of neuromodulation techniques such as deep brain stimulation or transcranial DC stimulation [20]. We believe that MREIT conductivity imaging will be of great use in providing *in vivo* conductivity information for biological tissues in what is known to be a physiologically relevant frequency range.

The delivery of imaging currents to the brain is difficult due to the low conductivity of skull bones. As a result, injected currents may induce a small phase signal, high noise level and low signal-to-noise-ratio (SNR) in brain tissue. Since the phase signals measured in MREIT may be quite small, SNR can be improved by increasing the imaging current amplitude or imaging time [8]. As in many other applications, intrinsic noise levels may be reduced by increased averaging or using higher field strengths. Therefore, in principle it should be possible to obtain sufficient SNR to observe brain tumors using MREIT, as long as imaging currents are applied for as long as possible, and if MR phase noise is low enough to allow averaging over a practical amount of time.

In this study, we are focused on the feasibility of applying MREIT to image *in vivo* brain tumors within the intact head. We approach this goal by constructing a finite element electromagnetic model of a realistically shaped human head, and simulating the effect of MREIT protocols with different sizes and locations of tumor conductivity changes. 

## 2. Methods

### 2.1. Three-Dimensional Head Model

**Figure 1:**
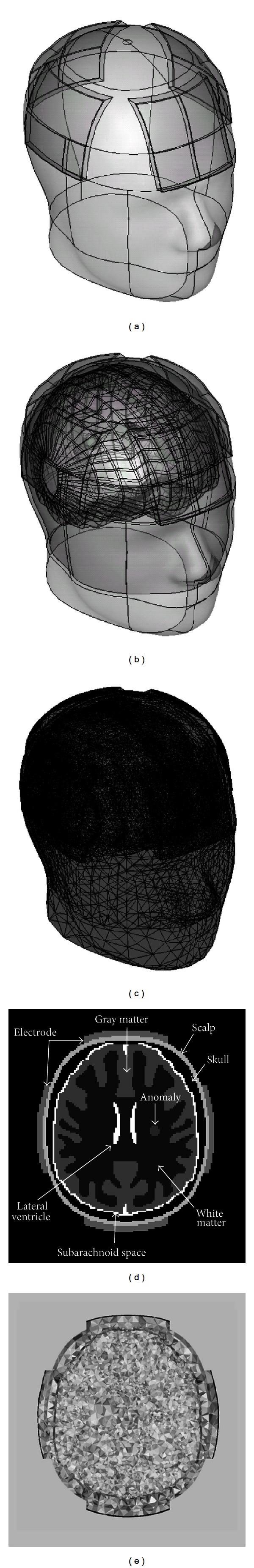
Overview of complete realistic head model. Shown here are (a) external geometry and electrode placement; (b) internal brain tissue; (c) completed mesh; (d) cross-sectional image of segmented structures, showing lateral ventricle, subarachnoid space, gray matter, white matter, skull, scalp, and 10 mm diameter tumor-like anomaly; and (e) cross-sectional image showing mesh quality at the same slice as shown in (d).

We built a three-dimensional finite element model based on a reference MRI data set consisting of 42 sagittal plane slices (3 mm thickness) over a 270 mm × 270 mm field of view (FOV) with an image matrix size of 512 × 512. Voxel sizes in the data set were therefore 0.53 mm × 0.53 mm × 3 mm. We used COMSOL (COMSOL Inc., Burlington, MA, USA) to extract the external head shape from the MRI data set. First, external contours of six transverse head projections were computed, then “lofted” together to form a three-dimensional solid structure. The resulting model had a volume of 4.2 L and a diameter at the temple of 17.5 cm. Four large MREIT electrodes (thicknesses 3 mm, area 64.5 cm^2^, and conductivity 0.17 S/m) were then added to the outer surface of the head (Figure 1(a)). Using the MRI data set as a guide, the head was further segmented into significant brain components: scalp, skull, gray matter (volume 0.4 L), white matter (volume 1.1 L), a subarachnoid layer (160 mL), and lateral ventricles (total volume 5.2 mL), as shown in Figures 1(b) and 1(c).

Conductivities used with the finite element model are shown in Table 1 [21–26]. Where possible, we chose recently measured values that were gathered in situations close to *in vivo* conditions. Values measured near 100 Hz were selected because MREIT currents are typically low frequency square waves (ca. 10–20 ms periods at 50% duty cycle). In our model, we assumed that white matter has isotropic conductivity of 0.058 S/m. Since the scalp consists of skin, muscle, a vascular layer, and fat, we considered an average conductivity value of 0.24 S/m to be reasonable. An isotropic conductivity of 0.0042 S/m was used for the skull [24]. There have been several studies on the electrical conductivity of the human cerebrospinal fluid [21]. We chose to use a value of 1.2 S/m in the subarachnoid space to most appropriately reflect its MREIT properties. This choice was made because of the small thickness of the component (approximately 2 mm, smaller than most voxels), and the mixture and proportions of tissues (bone, dura, CSF, and vessels) we expected to contribute to the properties of this region [22–26].

We included spherical anomalies of various diameters inside the brain component of the model to simulate tumors. The conductivity of these anomalies was chosen to be twice that of the surrounding normal brain tissues. In one version of the model, we introduced 8 spherical simple structured tumor-like anomalies, with diameters of 5, 7.5, 10, or 15 mm. In a second version of the model, we included 8 spherical complex structured anomalies consisting of angiogenic and necrotic tumor regions. The size of each region was half of the anomaly.

### 2.2. Numerical Simulation of Brain MREIT

The model was meshed into a large number (ca. 500000) of cubic tetrahedral finite elements as shown in Figure 1(c). In one head model containing a tumor-like anomaly, 448708 elements were created with a total number of degrees of freedom around 4.1 × 10^6^ (Figure 1(c)). The minimum element quality in the model was about 6.8 × 10^−3^ (Figure 1(e)). We solved for the Laplace equation in our model:

(1) $$\begin{array}{c} \nabla \cdot \left(\sigma \left(x,y,z\right)\nabla \phi \right)=0 \end{array}$$

on the head (Ω), subject to

(2) $$\begin{array}{c} \sigma \frac{\partial \phi }{dn}=j,\;\;\sum_{d\Omega }j=0, \end{array}$$

where *dΩ* is the head surface, *ϕ*  is the voltage distribution, *j* is the surface current density, and **n** is a vector normal to the surface. The quantity *σ*(*x*, *y*, *z*) is the conductivity distribution within the head. A total current of approximately 6.4 mA (a current density of 0.1 mA/cm^2^ underneath the electrode) was applied through each electrode in either left-right (LR) or anterior-posterior (AP) directions.

Voltage solutions were computed on the head domain, and then converted to magnetic flux density (*B*_*z*_) values within voxels of the size of 1.40 × 1.40 × 4 mm^3^ using the Biot-Savart law [7, 8] or a fast Fourier transform method [29]. Data were computed over a 180 × 180 mm^2^ field of view (FOV) and 8 slices in total were simulated, each slice having a thickness of 4 mm. The in-slice image matrix size was 128 × 128. Wires (length 2 cm, conductivity 20000 S/m) were connected to the center of each electrode, and at right angles to each electrode's surface to make the measurement more realistic. Further details of the simulation methods used in this paper may be found in Minhas et al. [29].

Reconstructions from *B*_*z*_ data to conductivity distributions at the selected resolution were performed using the harmonic *B*_*z*_ algorithm. This technique was first developed by Seo et al. [7, 15] and has been widely used in MREIT experiment studies. In this paper, conductivity reconstructions were performed using the CoReHA MREIT reconstruction package [28].

### 2.3. Noise Analysis in Brain Tumor Detection

We first examined the effect of introducing simple structured anomalies with 200% conductivity contrast with respect to the brain background in which it appeared. We then examined signal and reconstructed images resulting from a complex structured anomaly model having necrotic and angiogenic tumor regions. The noise standard deviation was derived from experimental measurements of noise in a clinical 3 T MRI system (Achieva TX, Philips Medical Systems, Best, The Netherlands) [30]. Noise was added to the simulated *B*_*z*_ voxel data based on

(3) $$\begin{array}{c} s_{}=\frac{1}{\sqrt{2}\gamma T_{c}Y_{M}}, \end{array}$$

where *Y*_*M*_ is the signal-to-noise ratio (SNR) in MR magnitude images, *γ*  is the gyromagnetic ratio of hydrogen (26.75 × 10^7^ radT^−1^s^−1^), and *T*_*c*_ is the injection current pulse duration. Since the *T*1, *T*2 value of gray, white matter and cerebrospinal fluid (CSF) are different [31, 32], the standard deviations of noise levels from different tissues were calculated separately and summed. Noise levels in each tissue were approximately 0.16 nT, 0.03 nT, and 0.013 nT with one excitation, respectively. Consider MRI acquisition at a voxel sized at Δ*x*, Δ*y*, and Δ*z* along the *x*,  *y*, and *z* directions, respectively. Let *N*_*x*_, *N*_*y*_, and *N*_*z*_ be the number of *k*-space samples in each of these directions, with a total readout sampling duration of *T*_*s*_ = *N*_*x*_ × Δ*t*, where Δ*t* is the time for one readout sample. Assuming that a spin-echo pulse sequence is used, the *TR* and *TE* dependence of the MR magnitude is given by

(4) $$\begin{array}{c} M=M_{0}\left(1-e^{-TR/T1}\right)e^{-TE/T2}, \end{array}$$

where *T*1 and *T*2 are spin relaxation times, and *M*_0_ is proton density. The magnitude image SNR, *Y*_*M*_, is given by the following relation [33]:

(5) $$\begin{array}{c} \Upsilon_{M}=M\Delta x\Delta y\Delta z\sqrt{N_{x}N_{y}N_{z}\Delta t\text{NEX}}. \end{array}$$

Substituting (5) into (3), we have

(6) $$\begin{array}{c} s=\frac{1}{\sqrt{2}\gamma^{}T_{c}M\Delta x\Delta y\Delta z\sqrt{N_{x}N_{y}N_{z}\Delta t^{}\text{NEX}}}. \end{array}$$

We now compare two different MREIT experiments performed with the same total scan time, same injection current amplitude, and same number of protons. If we denote the *B*_*z*_ noise standard deviations in each case to be *s*_0_ and *s*, respectively, then, substituting (4) into (6), we find that *s*_0_ and *s* are related by

(7) s=s0   ×(TcTc0ΔxΔx0ΔyΔy0ΔzΔz01−e−TR/T11−e−TR0/T10     ×e−TE/T2e−TE0/T20NNxNyNzΔtN0Nx0Ny0Nz0Δt0)−1.

The standard deviation of *B*_*z*_ noise levels expected in the human head was calculated by adjusting this figure using typical *TE* (time to echo) and *TR* (repetition time) values, and adjusting for the voxel size and number of averages selected. In this study, we used a 1000 ms *TR* and a *TE* of 30 ms.

## 3. Results

### 3.1. Simulation Results

**Figure 2:**
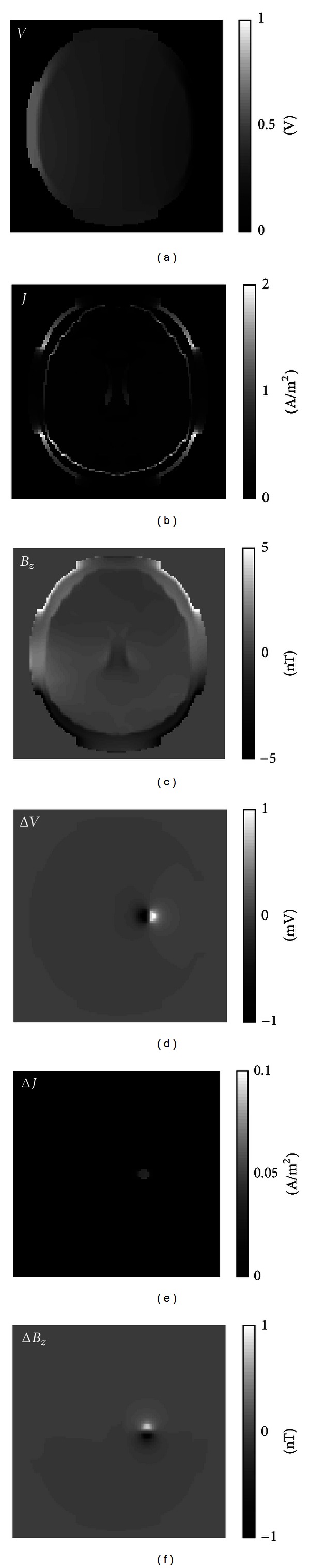
Axial slice of single anomaly model showing (a) voltage *V*, (b) current density magnitude *J*, and (c) magnetic flux density *B*_*z*_ values without anomaly present; and changes caused in the same slice with the anomaly having a 200% conductivity contrast from the brain background as (d) Δ*V*, (e) Δ*J*, and (f) Δ*B*_*z*_ distributions. Results are shown for LR current flow only. The injected current had 3 mA amplitude and a total current pulse width of 30 ms.

Figure 2 shows example data from the calculations with and without a single anomaly with 200% conductivity contrast for the case of 3 mA horizontal (LR) current and current application time *T*_*c*_ of 30 ms. The upper panels of the figure show plots of voltage (V), current density (A/m^2^), and magnetic flux density *B*_*z*_ (T), respectively, without the anomaly present. The lower panels show the changes in voltage, current density, and *B*_*z*_ that resulted when the tumor anomaly was introduced. The average current density value within the tumor was 0.035 A/m^2^, much lower than the value of 1.2 A/m^2^ that has been estimated as the threshold for neural excitation [34]. Changes in *B*_*z*_ due to the brain tumor were of the order of ±10^−10^ T in this case. We found that the anomaly perturbed the distributions of *V*,  *J*, and *B*_*z*_ and noted that the values of Δ*B*_*z*_ near the anomaly were greater than the noise level predicted in the measured *B*_*z*_ data. We found similar results for the second injection current *I*_2_. 

**Figure 3:**
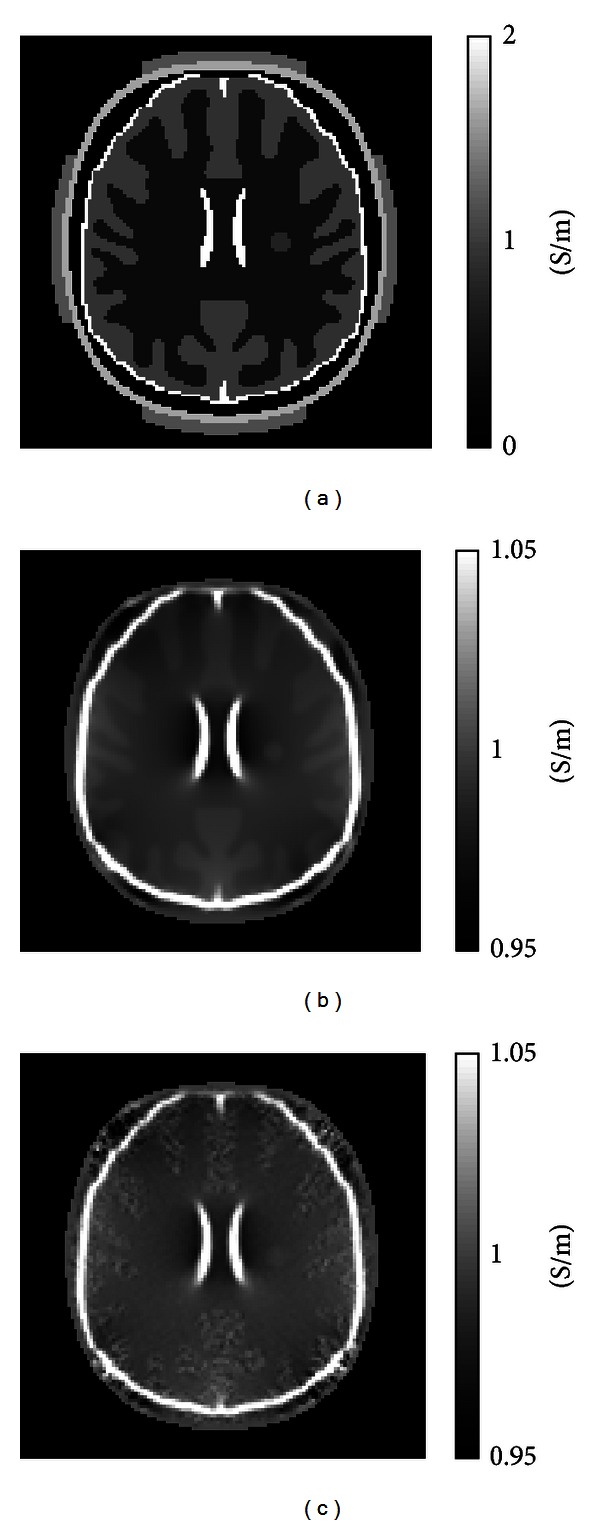
Conductivity images of brain model containing a single 10 mm diameter anomaly with 200% conductivity contrast. Image (a) shows the actual conductivity distribution. Images (b) and (c) show reconstructed conductivity images without and with noise, respectively. The injected current was 3 mA and the total current pulse width was 30 ms.

Reconstructed conductivity images using *B*_*z*_ data gathered from the single anomaly model in Figure 2 are shown in Figure 3.  Figure 3(a) shows the actual conductivity distribution, and Figures3(b) and 3(c) are reconstructed conductivity images created without and with experimental noise, respectively. To better simulate *in vivo* brain images, we added Gaussian noise with standard deviation values of 0.080 nT, and 0.016 nT, 0.007 nT (gray matter, white matter, and CSF) to simulate noise-contaminated *B*_*z*_ data generated with a number of averages of *N* = 4. As a result, reconstructed images of gray matter appear much noisier than white matter compartments overall. These values were computed using (7). Regardless of experimental noise, the MREIT reconstruction method could qualitatively differentiate tumor-like anomalies with diameters larger than 10 mm when the current amplitude of 3 mA was used. 

### 3.2. Brain Tumor Detection

**Figure 4:**
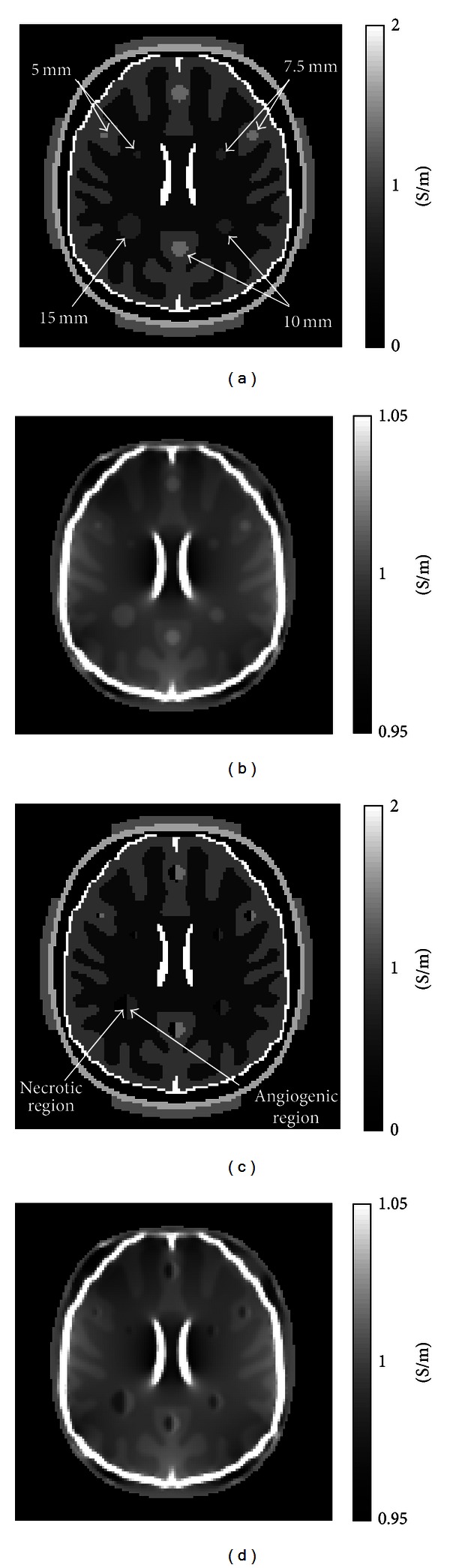
(a) Actual and (b) reconstructed conductivity images of 5, 7.5, 10 and 15 mm diameter anomalies having simple structures, using noise-free data. (c) and (d) are corresponding images of complex anomalies reconstructed using noise-free data.

To more comprehensively test the technique, we repeated the numerical simulations for the case of four different diameters of anomalies with 5, 7.5, 10, and 15 mm. Figures 4(a) and 4(b) show actual and reconstructed conductivity images of simple structure anomalies using noise-free *B*_*z*_ data. Because tumors can grow anywhere inside the brain, we placed anomalies inside both white and gray matter. Phase signals in anomalies near the boundaries of brain tissue had higher noise levels than those inside the brain. Figures 4(c) and 4(d) show images of complex structure anomalies we created to more realistically test our head model. Without noise, the MREIT reconstruction method could qualitatively differentiate tumor-like anomalies with diameters larger than the pixel size of 1.4 mm.

**Figure 5:**
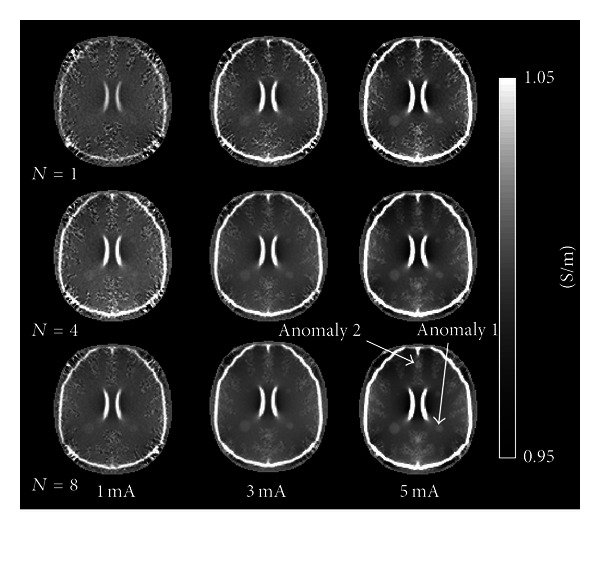
Reconstructed conductivity images of 5, 7.5, 10, and 15 mm diameter tumor-like anomalies having simple structures obtained using noisy data. Conductivity images were obtained at different numbers of averages *N* (top to bottom), and current amplitudes (left to right). Noise was added to the simulated *B*_*z*_ voxel data, based on experimental measurements of *B*_*z*_ noise in 3 T MR system.

Figure 5 shows reconstructed conductivity images of the simulated head model with simple structure anomalies created from data collected using different numbers of averages *N* and current amplitudes. The images represent reconstruction results with the NEX (the number of average *N*) increasing from 1 to 8 (top to bottom) and the amplitude of injected currents increasing from 1 to 5 mA (left to right). For any value of current amplitude, we can see that the image quality improves as we increase the number of averages *N*. Unfortunately, in the case of 1 mA imaging currents, even an anomaly having a 15 mm diameter could not be distinguished. For a fixed amount of noise in the *B*_*z*_ data (i.e., for a given value of *N*), we can improve image quality by increasing the current amplitude to produce *B*_*z*_ data with a larger dynamic range, that is, a higher SNR in the measured *B*_*z*_ data. For any value of *N*, and using either 3 or 5 mA injection currents, the 15 mm anomaly was clearly visible in reconstructions. In the white matter, the 10 and 7.5 mm anomalies were distinguishable at any value of *N*, using either 3 or 5 mA injection currents. The 5 mm anomaly was distinguishable when *N* = 4  and 8 with 5 mA injection current. In gray matter, anomalies having a diameter smaller than 10 mm were not clearly visible, even with the lowest noise level (*N* = 8, 5 mA injection current). The 10 mm anomaly was only partially distinguishable when *N* = 4 or 8 with 5 mA current.  Table 2 summarizes the standard deviations in conductivity values representing the improvement of the conductivity image in accordance with the different numbers of averages *N* and current amplitudes for simple structured anomalies.

**Figure 6:**
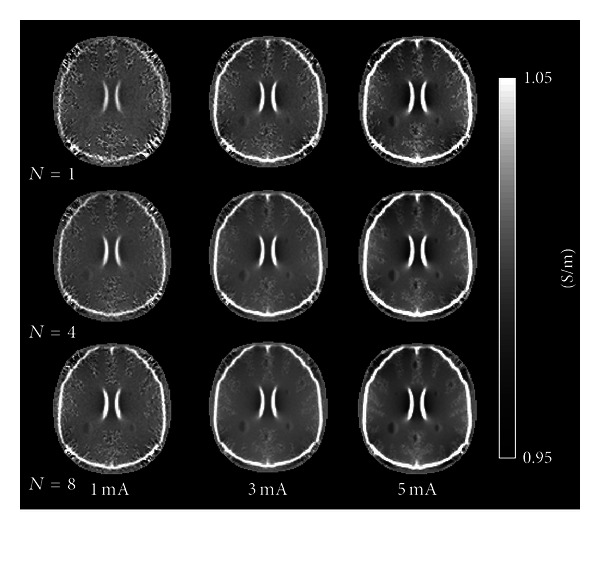
Reconstructed conductivity images of 5, 7.5, 10, and 15 mm diameter tumor-like anomalies having complex structures. The conductivity value of the angiogenic tumor region was two times higher than normal tissues and that of the necrotic region was 0.01 S/m. Conductivity images were obtained at different numbers of averages *N*  (top to bottom) and current amplitudes (left to right). Noise was added to the simulated *B*_*z*_ voxel data, based on experimental measurements of *B*_*z*_ noise in 3 T MR system.

Figure 6 shows the results of the same process applied to a complex structured anomaly model. As in the single anomaly cases, for 1 mA injection current, it was difficult to detect tumor-like anomalies when *N* = 1, 4, and 8, except for a 15 mm anomaly in white matter. As current amplitude was increased, some of the anomalies were detectable as *N* was progressively increased. In white matter, a 5 mm complex anomaly was not clearly visible even at *N* = 8 and 5 mA injection current. Interestingly, in 10 and 15 mm diameter tumors, the conductivity differences between necrotic and angiogenic tumor regions were easily distinguished. When *N* = 4 or 8 with 5 mA injection current, 7.5 mm anomalies also showed this pattern. Unfortunately, in gray matter, the 10 mm anomalies were clearly visible only at *N* = 4 or 8 with 5 mA current. The 7.5 mm anomaly was detectable but did not display a conductivity contrast between the necrotic and angiogenic regions of the tumor.

### 3.3. Analysis of Δ*B*_*z*_ with Noise Level

**Figure 7:**
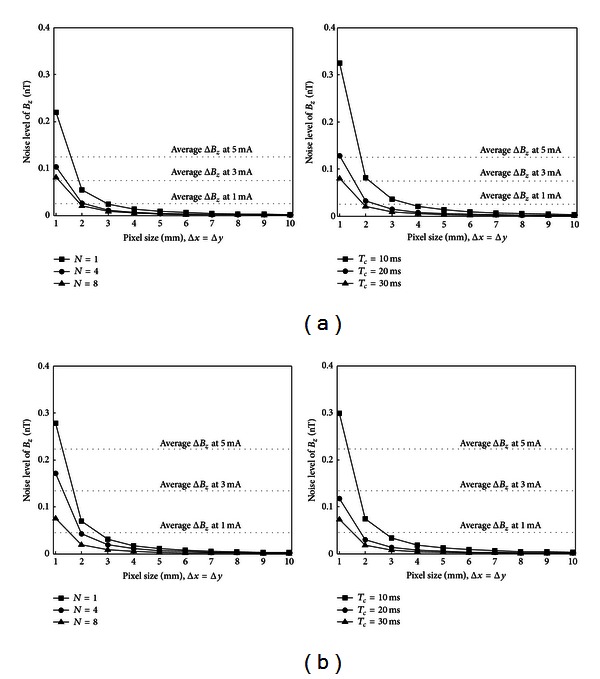
Nomogram showing predicted *B*_*z*_ noise levels compared to the estimated signal size at different pixel sizes using a fixed slice thickness of 4 mm and assuming a 10 mm anomaly diameter in both (a) white and (b) gray matter. Three different values of averaging NEX at *T*_*c*_ of 30 ms and three different values of total current injection time *T*_*c*_ at *N* = 8 were used. The values of average Δ*B*_*z*_ are compared for a simulated conductivity contrast of 200% and for an anomaly diameter of 10 mm.

For a tumor-like anomaly to be detected inside the brain, the change in the measured *B*_*z*_, due to the presence of an anomaly, Δ*B*_*z*_, must be larger than the noise level in measured *B*_*z*_ data. Δ*B*_*z*_ values are influenced by the injection current amplitude and anomaly size. We computed average values of Δ*B*_*z*_ inside anomaly regions, and compared them with noise levels *s*, which in turn are determined by imaging parameters and tissue properties of *T*1 and *T*2 as shown in (7). Figures 7(a) and 7(b) illustrate how the noise level *s* in measured *B*_*z*_ should change with pixel size Δ*x* = Δ*y* as the number of averages *N* and the total current injection time *T*_*c*_ are varied in both white and gray matter. In both cases, the slice thickness Δ*z* was 4 mm and the diameter of the anomaly was 10 mm. These plots provide a useful guide for the selection of parameters such as Δ*x*,  Δ*y*,  *N*, and *T*_*c*_ and the injection current amplitude required to produce *B*_*z*_ data that are capable of visualizing a certain anomaly in the presence of a particular estimated noise level. We may easily scale up and down the plots in Figure 7 for different situations.

## 4. Discussion

We have demonstrated that tumor-like anomalies with 200% conductivity contrast can straightforwardly be both detected and imaged by an existing 3 T system using total acquisition times below 30 minutes. Smaller anomalies (ca. 5 mm diameter) could not easily be discerned in images, even with 8 averages. However, it may be possible to detect these smaller anomalies when we alter imaging parameters further, such as by increasing the number of averages above 8, or increasing the image resolution, but this will of course increase overall averaging time. The 7.5, 10, and 15 mm diameter anomalies with 200% contrast were detectable in white matter, but the 10 mm gray matter anomaly was only visible at the lowest relative noise level. In the complex anomaly cases, the boundaries between the low-conductivity necrotic region and high-conductivity angiogenic regions were clearly contrasted in 10 and 15 mm diameter anomalies. These results may provide evidence that MREIT can be used not only to detect brain tumors, but may also provide useful tumor characterization information.

The model we have used here is detailed with respect to the principal conductivity contrasts within the head. Those critical to delivering the current to the head are scalp and skull conductivity, as well as CSF conductivity [20]. There are several improvements that could be made to our model, including differentiation of both cortical and cancellous bone, and the use of anisotropic conductivity in white matter. These could cause some modification to the current density distribution and therefore to the predicted signal levels [35]. Another modification that we have included here is to differentiate between peripheral and ventricular CSF. MR data is intrinsically volumetric and therefore data in one voxel is averaged over all tissues within. The layer between the skull and cortex is very thin and made up of many different components, only one of which is CSF. Therefore, we estimated the value for conductivity in this area as a mix of CSF and other tissues not modeled, including the dura, skull, and blood vessels. The consideration of the model's construct validity extends to the inclusion of the expected conductivity change. While few estimates of the size of conductivity changes to be expected from brain tumors have been made, we selected the values that have been most widely used. Further modeling and experimentation will be guided by improvements in these basic measurements.

The *B*_*z*_ data in this study were collected using a conventional, simple spin echo MR sequence. More sensitive and faster pulse sequences are currently in development [8, 36] and may further improve phase data, signal-to-noise-ratio (SNR), and sensitivity. The use of multiple RF coils and newer, lower-noise systems should further enhance SNR.

The harmonic *B*_*z*_ algorithm implemented in CoReHA produces conductivity images with relative conductivity contrasts [28]. A variation of the harmonic *B*_*z*_ algorithm known as the local harmonic *B*_*z*_ algorithm (LBz) can be used to perform conductivity data within a specified region of interest [16, 17]. This approach has the benefit of avoiding reconstructions using data where SNR is particularly low. Additionally, denoising steps can greatly improve the quality of reconstructed images [37, 38]. While absolute conductivity images are advantageous in imaging tumors or other static physiological presentations, conductivity contrast images should be sufficient for most MREIT applications.

While we believe that this study shows that MREIT is realizable in human brain tumor detection, there are other considerations that must be taken into account when performing *in vivo* imaging. First, we are unsure of the extent to which physiological, and in particular hemodynamic-changes should affect signal-to-noise ratios. Only *in vivo* testing will allow us to determine the effect of these signals. We have shown that MREIT should be capable of imaging realistically sized brain tumor conductivity changes occurring within gray matter regions using conventional MR systems and over a practical period of time. Testing* in vivo* will allow us to determine the extent and size of physiological changes.

## 5. Conclusion

In this study, we have shown the feasibility of MREIT conductivity imaging for brain tumor detection. We simulated the effect of MREIT protocols with different sizes and locations of conductivity change, using a finite element electromagnetic model of a human head. Conductivity values used in our models were taken from well-accepted and recent sources, with attention paid to compartment environments. As well as modeling the skull compartment conductivity accurately, it is also important in simulation studies to use realistic skull and head geometries and to appropriately segment the head model. To better apply this technique *in vivo*, advanced head MR imaging methods including pulse sequence (such as GRE, EPI, and SSFP), *k*-space sampling strategies, and multichannel high-sensitivity RF coils may be employed to minimize the noise level in measured magnetic flux density data and thus reduce the current and time needed to produce good conductivity resolution. 

## Figures and Tables

**Table 1:** Conductivities used in finite element models, with sources.

Component	Conductivity (S/m)	Comments/sources
Gray Matter	0.09	Gabriel et al. [22]
White Matter	0.06	Gabriel et al. [22]
CSF (ventricle)	1.80	Baumann et al. [21]
Subarachnoid space	1.20	Estimated from relative contributions of dura 0.5, CSF 1.8, skull 0.02, blood 0.67, and vessel 0.26 S/m per Gabriel et al. [22]
Skull	0.0042	Dannhauer et al. [24]
Scalp	0.24	Estimated from relative contributions of muscle 0.27, skin 0.00046, blood 0.67, vessel 0.26, and fat 0.02 S/m per Gabriel et al. [22]
Tumor-like anomaly in gray matter	0.20	2 times increase over gray matter value
Tumor-like anomaly in white matter	0.12	2 times increase over white matter value
Necrotic region	0.01	Oh et al. [27]
Hydrogel electrode	0.17	Jeon et al. [28]

**Table 2:** Measured standard deviation of reconstructed conductivity from Figure 5. ROI (region of interest) was located in the normal gray, white matter, and anomalies with the voxel size of 5 × 5 × 5 mm^3^. Note CoReHA provides only conductivity contrast information. We therefore show standard deviation of conductivity values as a quantitative criterion representing an improvement in conductivity images.

Current	Measured standard deviation in conductivity (mS/m)								
	White (gray) matter			Anomaly 1			Anomaly 2		
	1 NEX	4 NEX	8 NEX	1 NEX	4 NEX	8 NEX	1 NEX	4 NEX	8 NEX
1 mA	1.4 (6.3)	0.9 (3.1)	0.6 (2.6)	1.3	0.6	0.4	5.4	2.7	2.4
3 mA	1.1 (5.9)	0.6 (2.4)	0.4 (2.1)	0.8	0.4	0.3	5.2	2.4	2.0
5 mA	0.9 (4.7)	0.4 (1.9)	0.3 (1.4)	0.6	0.4	0.2	3.2	2.3	1.9

## References

[1] Moffat BA, Chenevert TL, Lawrence TS (2005). Functional diffusion map: a noninvasive MRI biomarker for early stratification of clinical brain tumor response. *Proceedings of the National Academy of Sciences of the United States of America*.

[2] Van de Wiele C, Lahorte C, Oyen W (2003). Nuclear medicine imaging to predict response to radiotherapy: a review. *International Journal of Radiation Oncology Biology Physics*.

[3] Kaplan WD, Takvorian T, Morris JH (1987). Thallium-201 brain tumor imaging: a comparative study with pathologic correlation. *Journal of Nuclear Medicine*.

[4] Barajas RF, Cha S (2012). Imaging diagnosis of brain metastasis. *Progress in Neurological Surgery*.

[5] Young GS (2007). Advanced MRI of adult brain tumors. *Neurologic Clinics*.

[6] Oh SH, Lee BI, Woo EJ (2003). Conductivity and current density image reconstruction using harmonic Bz algorithm in magnetic resonance electrical impedence tomography. *Physics in Medicine and Biology*.

[7] Woo EJ, Seo JK (2008). Magnetic resonance electrical impedance tomography (MREIT) for high-resolution conductivity imaging. *Physiological Measurement*.

[8] Minhas AS, Jeong WC, Kim YT, Han YQ, Kim HJ, Woo EJ (2011). Experimental performance evaluation of multi-echo ICNE pulse sequence in magnetic resonance electrical impedance tomography. *Magnetic Resonance in Medicine*.

[9] Joy M, Scott G, Henkelman M (1989). *In vivo* detection of applied electric currents by magnetic resonance imaging. *Magnetic Resonance Imaging*.

[10] Scott GC, Joy MLG, Armstrong RL, Henkelman RM (1991). Measurement of nonuniform current density by magnetic resonance. *IEEE Transactions on Medical Imaging*.

[11] Scott GC, Joy MLG, Armstrong RL, Henkelman RM (1992). Sensitivity of magnetic-resonance current-density imaging. *Journal of Magnetic Resonance*.

[12] Park C, Kwon O, Woo EJ, Seo JK (2004). Electrical conductivity imaging using gradient *B*
_*z*_ decomposition algorithm in magnetic resonance electrical impedance tomography (MREIT). *IEEE Transactions on Medical Imaging*.

[13] Gao N, Zhu SA, He B (2006). A new magnetic resonance electrical impedance tomography (MREIT) algorithm: the RSM-MREIT algorithm with applications to estimation of human head conductivity. *Physics in Medicine and Biology*.

[14] Birgül O, Eyüboğlu BM, Ider YZ (2003). Experimental results for 2D magnetic resonance electrical impedance tomography (MR-EIT) using magnetic flux density in one direction. *Physics in Medicine and Biology*.

[15] Seo JK, Yoon JR, Woo EJ, Kwon O (2003). Reconstruction of conductivity and current density images using only one component of magnetic field measurements. *IEEE Transactions on Biomedical Engineering*.

[16] Kim HJ, Lee BI, Cho Y (2007). Conductivity imaging of canine brain using a 3 T MREIT system: postmortem experiments. *Physiological Measurement*.

[17] Kim HJ, Oh TI, Kim YT (2008). In vivo electrical conductivity imaging of a canine brain using a 3 T MREIT system. *Physiological Measurement*.

[18] Kim HJ, Kim YT, Minhas AS (2009). In vivo high-resolutionconductivity imaging of the human leg using MREIT: the first human experiment. *IEEE Transactions on Medical Imaging*.

[19] Sadleir RJ, Grant SC, Woo EJ (2010). Can high-field MREIT be used to directly detect neural activity? Theoretical considerations. *NeuroImage*.

[20] Sadleir RJ, Vannorsdall TD, Schretlen DJ, Gordon B (2010). Transcranial direct current stimulation (tDCS) in a realistic head model. *NeuroImage*.

[21] Baumann SB, Wozny DR, Kelly SK, Meno FM (1997). The electrical conductivity of human cerebrospinal fluid at body temperature. *IEEE Transactions on Biomedical Engineering*.

[22] Gabriel C, Gabriel S, Corthout E (1996). The dielectric properties of biological tissues: I. Literature survey. *Physics in Medicine and Biology*.

[23] Akhtari M, Bryant HC, Mamelak AN (2000). Conductivities of three-layer human skull. *Brain Topography*.

[24] Dannhauer M, Lanfer B, Wolters CH, Knosche TR (2011). Modeling of the human skull in EEG source analysis. *Human Brain Mapping*.

[25] Oostendorp TF, Delbeke J, Stegeman DF (2000). The conductivity of the human skull: results of in vivo and in vitro measurements. *IEEE Transactions on Biomedical Engineering*.

[26] Latikka J, Kuurne T, Eskola H (2001). Conductivity of living intracranial tissues. *Physics in Medicine and Biology*.

[27] Oh TI, Jeong WC, McEwan A Feasibility of MREIT conductivity imaging to evaluate brain abscess lesion: in vivo canine model.

[28] Jeon K, Minhas AS, Kim YT (2009). MREIT conductivity imaging of the postmortem canine abdomen using CoReHA. *Physiological Measurement*.

[29] Minhas AS, Kim HH, Meng ZJ, Kim YT, Kim HJ, Woo EJ (2011). Three-dimensional MREIT simulator of static bioelectromagnetism and MRI. *Biomedical Engineering Letters*.

[30] Sadleir R, Grant S, Sung UZ (2005). Noise analysis in magnetic resonance electrical impedance tomography at 3 and 11 T field strengths. *Physiological Measurement*.

[31] Stanisz GJ, Odrobina EE, Pun J (2005). T1, T2 relaxation and magnetization transfer in tissue at 3T. *Magnetic Resonance in Medicine*.

[32] Schmitt P, Griswold MA, Jakob PM (2004). Inversion Recovery TrueFISP: quantification of *T*
_1_, *T*
_2_, and Spin Density. *Magnetic Resonance in Medicine*.

[33] Haacke EM, Brown RW, Thompson MR, Venkatesan R (1999). *Magnetic Resonance Imaging: Physical Principles and Sequence Design*.

[34] Reilly JP (1998). *Applied Bioelectricity, High-Voltage and High-Current Injuries*.

[35] Sadleir RJ, Argibay A (2007). Modeling skull electrical properties. *Annals of Biomedical Engineering*.

[36] Nam HS, Kwon OI (2010). Optimization of multiply acquired magnetic flux density *B*
_*z*_ using ICNE-multiecho train in MREIT. *Physics in Medicine and Biology*.

[37] Lee BI, Lee SH, Kim TS, Kwon O, Woo EJ, Seo JK (2005). Harmonic decomposition in PDE-based denoising technique for magnetic resonance electrical impedance tomography. *IEEE Transactions on Biomedical Engineering*.

[38] Lee CO, Ahn S, Jeon K (2009). *Denoising of Bz Data for Conductivity Reconstruction in Magnetic Resonance Electrical Impedance Tomography (MREIT)*.

